# A Cell’s Viscoelasticity Measurement Method Based on the Spheroidization Process of Non-Spherical Shaped Cell

**DOI:** 10.3390/s21165561

**Published:** 2021-08-18

**Authors:** Yaowei Liu, Yujie Zhang, Maosheng Cui, Xiangfei Zhao, Mingzhu Sun, Xin Zhao

**Affiliations:** 1 Institute of Robotics and Automatic Information System, Tianjin Key Laboratory of Intelligent Robotics, Nankai University, Tianjin 300071, China; liuyaowei@mail.nankai.edu.cn (Y.L.); zhangyujie1002@mail.nankai.edu.cn (Y.Z.); 1120170124@mail.nankai.edu.cn (X.Z.); sunmz@nankai.edu.cn (M.S.); 2 Institute of Animal Sciences, Tianjin 300112, China; tjsnykxyxmsyyjs@tj.gov.cn

**Keywords:** robotic cell manipulation, mechanical properties, elasticity measurement, viscosity measurement, cell mechanics

## Abstract

The mechanical properties of biological cells, especially the elastic modulus and viscosity of cells, have been identified to reflect cell viability and cell states. The existing measuring techniques need additional equipment or operation condition. This paper presents a cell’s viscoelasticity measurement method based on the spheroidization process of non-spherical shaped cell. The viscoelasticity of porcine fetal fibroblast was measured. Firstly, we introduced the process of recording the spheroidization process of porcine fetal fibroblast. Secondly, we built the viscoelastic model for simulating a cell’s spheroidization process. Then, we simulated the spheroidization process of porcine fetal fibroblast and got the simulated spheroidization process. By identifying the parameters in the viscoelastic model, we got the elasticity (500 Pa) and viscosity (10 Pa·s) of porcine fetal fibroblast. The results showed that the magnitude of the elasticity and viscosity were in agreement with those measured by traditional method. To verify the accuracy of the proposed method, we imitated the spheroidization process with silicone oil, a kind of viscous and uniform liquid with determined viscosity. We did the silicone oil’s spheroidization experiment and simulated this process. The simulation results also fitted the experimental results well.

## 1. Introduction

The mechanical properties of biological cells, especially the elastic modulus and viscosity of cells, can provide an important basis for the evaluation of cell viability and cell states and the judgment of biological activity [1,2,3], and is crucial for the understanding of cell structure and physiological function [4,5,6]. To measure cell viscoelasticity, scientists have developed methods such as atomic force microscopy (AFM) [7,8,9,10,11,12], magnetic tweezers technique [13,14], optical tweezers technique [15,16], microfluidic technique [17,18,19], and micropipette aspiration (MA) technique [20,21,22,23,24]. These methods are suitable for different situations. AFM technique detects the viscoelasticity of the cell by moving the cantilever probe in vertical direction and monitoring its bending displacement. Magnetic tweezers technique and optical tweezers technique apply a certain force to the magnetic beads or silicon beads adhered to cells through magnetic field or light field to deform cells and obtain the viscoelasticity of cells. Microfluidic technique obtains the viscoelasticity of cells by detecting the deformation of cells under different microchannels and different shear forces. MA technique obtains the viscoelasticity of cells by measuring the length of the cells aspirated into the micropipette under different pressures.

Among these techniques, MA technique has become widely used due to the reasons of no need to purchase or prepare additional equipment, lower measurement cost, and easier integration into existing commercial micro-operation systems [25]. However, the micropipette aspiration method has high requirements for the seal between the cell and the micropipette in the measurement process [26]. Slightly improper sealing will result in ineffective MA operations, which will have a great impact on the measurement results. Meanwhile, the measuring of results is highly dependent on the accuracy of the force sensor. The viscoelasticity differences of the same cells measured by the same research groups using the micropipette aspiration method will also be very large. For example, the elasticity of human chondrocytes measured by Jones et al. was 0.65 ± 0.63 kPa [27], wherein the standard deviation was as large as the measured value. As the shape of the cell might be non-spherical, it will be more difficult to seal the cell and micropipette. In order to eliminate the influence of sealing on the measurement results, it is necessary to design a cell viscoelastic measurement method based on the micropipette aspiration platform and with low requirements for sealing.

**Figure 1:**
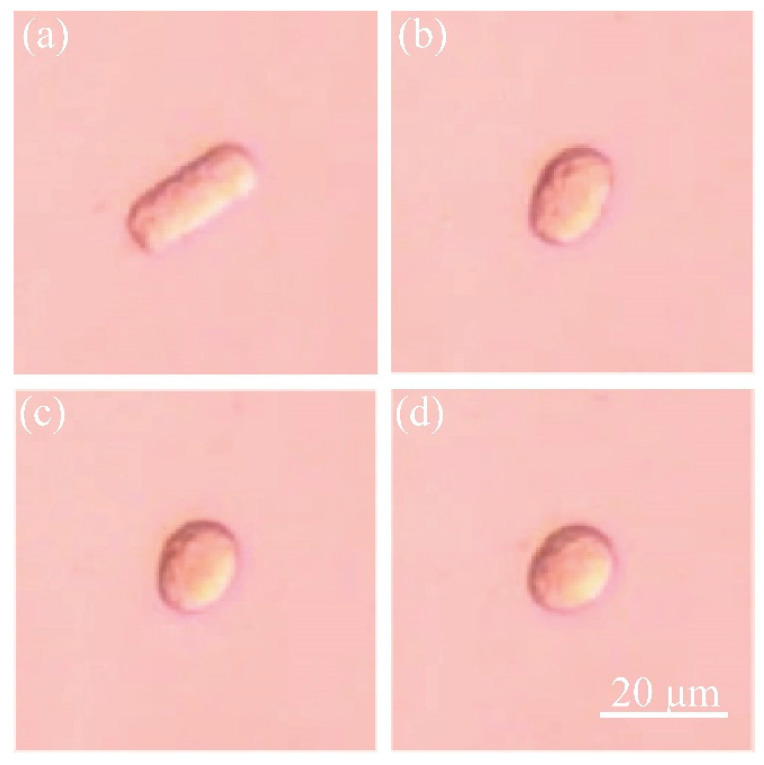
Spheroidization process of the capsule-like porcine fetal fibroblast. (**a**–**d**) From capsule-like cell to spherical-like cell.

In this paper, we proposed a cell’s viscoelasticity measurement method based on the spheroidization process of non-spherical shaped cell. The spheroidization process means the process of some deformable non-spherical objects turning into spherical shapes due to surface tension. We firstly introduced the method of recording the spheroidization process of porcine fetal fibroblast and recorded the fetal fibroblast’s spheroidization process. Secondly, we built the viscoelastic model for simulating non-spherical shaped cell’s spheroidization process based on the fact that the capsule-like porcine fetal fibroblast will finally become spherical (Figure 1). Then, we simulated the spheroidization process of porcine fetal fibroblast and got the simulated spheroidization process. By changing the parameters in the simulations, we got the elasticity and viscosity that best fitted the experiments. The magnitude of the elasticity and viscosity of fetal fibroblast was in agreement with those measured in other literatures. To verify the accuracy of this method, we imitated the spheroidization process with silicone oil, a kind of viscous and uniform liquid with determined viscosity. We did the silicone oil’s spheroidization experiment and simulated this process. The simulation results fitted the experimental results well.

## 2. Materials and Methods

### 2.1. System Setup

**Figure 2:**
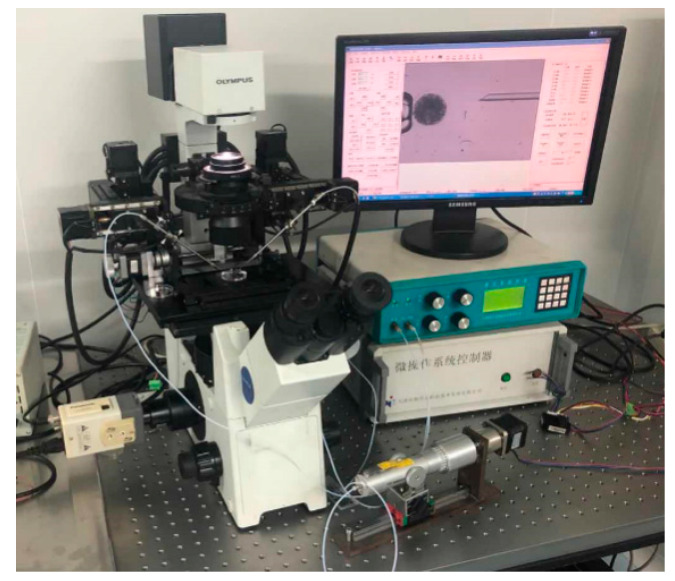
NK-MR601 micro-operation system.

The spheroidization experiment of porcine fetal fibroblast was performed on the self-developed NK-MR601 micro-operation system [28,29,30] (Figure 2). The system consists a microscope (CK-40, Olympus, Tokyo, Japan); a CCD camera (W-V-460, Panasonic, Osaka, Japan, frame rate: 20 frame/s); a motorized X-Y stage (travel range: 100 mm, repeatability: ±1 μm/s, maximum speed: 2 mm/s); two X-Y-Z manipulators (travel range: 50 mm, repeatability: ±1 μm/s, maximum speed: 1 mm/s); a self-developed micro-injector, providing negative pressure to aspirate the fetal fibroblast and positive pressure to eject the fetal fibroblast; a self-developed motion control box, controlling the micro-platform, micro-manipulators, and micro-injector through the host computer.

The silicone oil spheroidization experiment was performed on NK-MR601 with the CCD replaced by a highspeed camera (C110, Miro, Wayne, NJ, USA, frame rate: 1000 frame/s).

The micropipettes used in the spheroidization experiments of porcine fetal fibroblast and silicone oil were made from borosilicate glass tubes with an outer diameter of 1 mm and an inner diameter of 0.8 mm. The micropipette used in the fetal fibroblast spheroidization experiments were pulled by the puller (MODEL P-97, Sutter Instrument, Novato, CA, USA), and fractured by the microforge (MF-900, NARISHIGE, Tokyo, Japan) with an inner diameter of 10 μm. The micropipette used in the silicone oil spheroidization experiments was pulled and fractured by hand by Yaowei Liu, with an outer diameter of 200 μm.

### 2.2. Preparation and Spheroidization of Porcine Fetal Fibroblast

We obtained the porcine fetal fibroblast from a sow at day 35 of pregnancy. After removal of head, internal organs and limbs, the remaining parts were cut into pieces at approximately 1 mm^3^. We smeared the pieces evenly in a 35 mm dish and cultured in Dulbecco’s modified Eagle’s medium (DMEM), containing 15% fetal calf serum (FCS), 0.1 mM non-essential amino acids (NEAA), 6 μL/mL Gentamycin and 0.05 mM L-glutamine. Cells were cultured in a 37 °C humidified incubator containing 5% CO_2_. Cells were trypsinized and cryo-preserved for use when cells grown to ~90% confluence.

**Figure 3:**
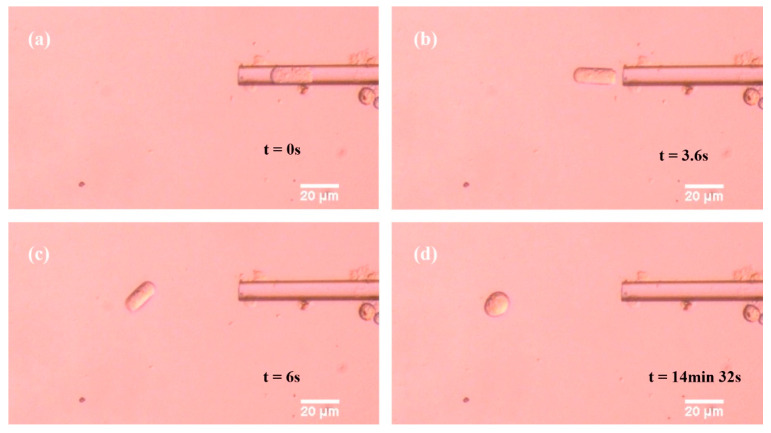
Typical images in the spheroidization process of porcine fetal fibroblast. (**a**–**d**) From porcine fetal fibroblast just coming out of the micropipette to porcine fetal fibroblast becoming a sphere.

The spheroidization experiments of porcine fetal fibroblast were carried out in Medium 199 (Sigma). Figure 3 shows the typical images of the porcine fetal fibroblast spheroidization process:

(1) Give negative pressure in the micropipette to aspirate the cell into the micropipette;
(2) Give positive pressure in the micropipette to eject the capsule-like porcine fetal fibroblast out of the micropipette;
(3) Record the length and the width of the non-spherical shaped cell;
(4) The end of the spheroidization process. The pressure was adjusted by hand. The cells were placed near the tip of micropipette initially and aspirated into the micropipette for more than 10 s. The images were captured with 50 frames per second and measured with 2 frames per minute. The initial ratio was determined by the inner diameter of micropipette and the cell volume in the experiment. The method of detecting the size of capsule-like fetal fibroblast is described in Appendix A.

### 2.3. Spheroidization of Silicone Oil

**Figure 4:**
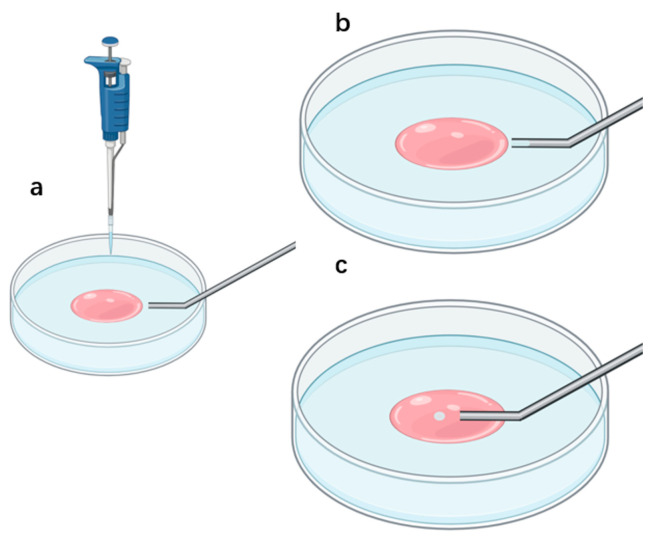
(**a**) Drop M199 into a petri dish and overlay M199 with silicone oil; (**b**) aspirate silicone oil into the micropipette; (**c**) move the micropipette tip into M199 and eject silicone oil.

Figure 4 shows the method of recording the silicone oil spheroidization process:

(1) Drop culture medium M199 (Sigma) into a petri dish (Corning, 430165 35 mm × 10 mm). Overlay M199 drop with silicone oil (Sigma-Aldrich, St. Louis, MO, USA). The pink liquid in Figure 4 represents M199 and the blue liquid represents the silicone oil.
(2) Move the micropipette tip into the silicone oil drop. Give negative pressure in the micropipette to aspirate some silicone oil into the micropipette.
(3) Move the micropipette tip into M199 solution. Provide positive pressure in the micropipette to eject silicone into M199 solution. Record the silicone oil spheroidization process with a high-speed camera.

### 2.4. Viscoelastic Model

We use a viscoelastic model to study the spheroidization process. The cell is modeled as homogeneous viscoelastic liquid, which is surrounded by infinitesimal thin cortical layer. We use the Jeffrey’s viscoelastic fluid model (Equation (2)) because it is independent of the frame of reference and the motion as a whole in space [31]. Besides, it has only 2 additional parameters, while being able to imitate the viscoelastic behavior. More complex models (e.g., heterogeneous liquid) are hard to modify the parameters to obtain reliable results. In the simulation, the cortical layer is realized by surface tension. We made the following assumptions:

(1) The inner material of fibroblast is homogeneous and isotropic. Based on this assumption we can get global cell properties.
(2) The fibroblast is incompressible. It is for the ease of simulation.
(3) The influence of gravity and pressure variance because of different depth is negligible. It is reasonable by comparing the gravity and pressure variance with hydrostatic pressure (about 1/10^6^ in micron scale).

(1) $$\overset{\nabla }{T}=\frac{\partial T}{\partial T}+(v\cdot \nabla )T-\nabla v\cdot T-T\cdot {\left(\nabla v\right)}^{T}$$

(2) $$\lambda \overset{\nabla }{T}+T=2\eta E$$

(3) $$\rho \frac{Dv}{Dt}=\nabla \cdot (-pI+K+T)$$

(4) K=2μE

where $\overset{\nabla }{T}$ denotes the upper convection derivative [32] of ***T*** defined by Equation (1). ***T*** is the viscoelastic stress tensor that changes with time according to Equation (2). ***v*** is the velocity field. *λ* is the characteristic time. *η* is the viscosity in the viscoelastic term. ***E*** is the strain-rate tensor. *ρ* is the density of porcine fetal fibroblast and is assumed to be constant in the following simulation. *p* is the pressure. *D****v***/*Dt* is the material derivative of ***v***. **I** is the unit tensor. ***K*** is the shear stress tensor which can be obtained from Equation (4). *μ* is another viscosity in the pure viscous term. Equation (3) is the Navier-Stokes equation.

**Figure 5:**
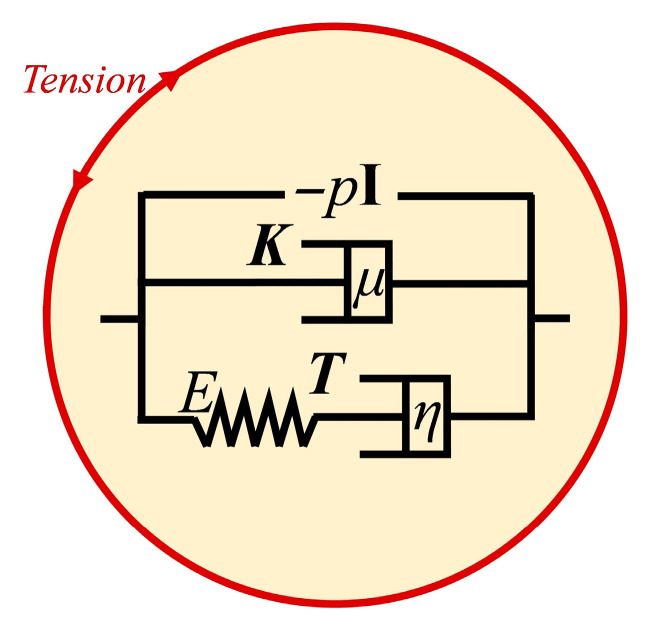
Illustration of viscoelastic model.

An analogy of the model in one dimension can be illustrated as Figure 5. The total stress tensor (right terms in the bracket of Equation (3)) is composed of hydrostatic pressure −*p***I**, viscous stress ***K*** and viscoelastic stress ***T***. In this figure, *E* is the stiffness coefficient of spring, *η* and *μ* are the viscosities of two dashpots, and λ=η/E. The bottom line represents a Maxwell model, for which the relationship of strain rate *e* and stress *σ* is $\lambda \dot{\sigma }+\sigma =\eta e$ in 1D case. By replacing the time derivative with upper convected derivative and extend the equation to 3D tensor form, we get Equation (2). The usage of upper convected derivative for continuum materials was argued by Oldroyd in [31].

### 2.5. Simulation of the Spheroidization Process

We used Ansys Student Fluent software to simulate the spheroidization processes. Two-dimensional axisymmetric was adopted for efficiency.

**Figure 6:**
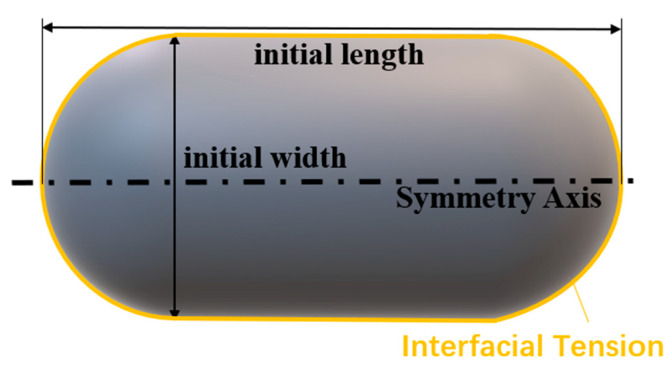
Capsule-like porcine fetal fibroblast.

For the simulation of fibroblast, we set the capsule-like porcine fetal fibroblast as a cylinder in the middle and two hemispheres at both ends, as shown in Figure 6 (only 1/4 part was used by applying the axisymmetric and symmetric condition). In this paper, the length was set as 20.8 μm, the width was set as 8.6 μm. The computation domain was a rectangle of 16 × 10 μm^2^ which was divided into 0.1 × 0.1 μm^2^ structural quadrilateral grids. The pressure variance in the scale of several micrometers is of the order of 0.01 Pa, which is far less than the barometric pressure. Besides, it is balanced by the gravity, so we neglected both pressure variance and the gravity. We used volume of fluid (VOF) model to introduce the surface tension. Laminar flow was adopted because of low Reynolds number. We set the four boundaries as axisymmetric, symmetric and pressure outlet, respectively. The densities were set as 1080 kg/m^3^ [25] for fibroblast and 998.2 kg/m^3^ (the density of water at 20 °C) for surrounding liquid. The surface tension coefficient *T* was set as 10 μN/m [33]. The viscosity *η* and elasticity *E* (*E* = *η*/*λ*) were introduced with user-defined scalars (UDS, see Appendix B). Ansys Student Fluent software solves the momentum Equation (3) without viscoelastic stress term ***T*** by default. We used user-defined scalars (UDS) to insert ***T*** into the equation (see Appendix B for more details). To study the influence of viscosity and elasticity, we firstly set *λ* = 1 s, and changed viscosity *η* as 10 Pa·s, 20 Pa·s, 50 Pa·s, 100 Pa·s, 200 Pa·s, and 500 Pa·s. Secondly, we set *η* = 500 Pa·s, and changed *λ* as 500 s, 100 s, 20 s, 1 s, 0 s, 1 s and 0.02 s [33]. The timestep was 3 s. Based on the results obtained when the parameters selected in a wide range, we made more compact selections and compared the results with experimental data. The one that fitted best was viewed as measurement result.

**Figure 7:**
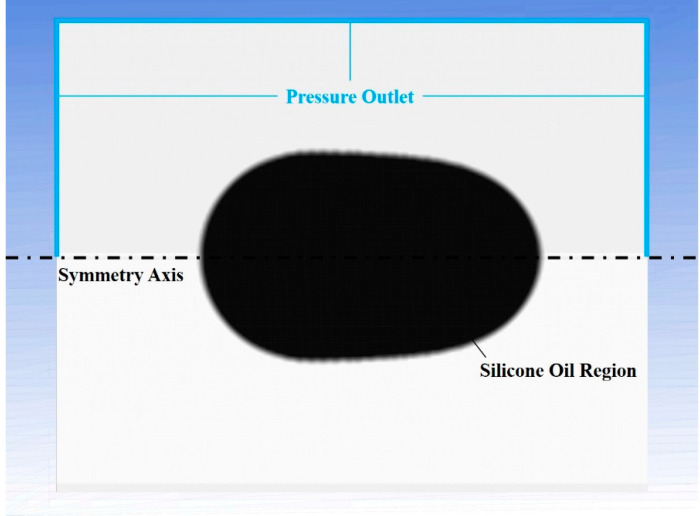
Geometric shape and boundary settings.

For the simulation of silicone oil, the initial shape was set as axisymmetric while the contour being obtained by image processing procedure (see Appendix A). The computation domain was a rectangle of 500 × 200 μm^2^ which was divided into 2 × 2 μm^2^ structural quadrilateral grids. As is shown in Figure 7, one boundary is symmetric axis and others are pressure outlet. Using the contour obtained by image processing (Appendix A), a user-defined function sets the corresponding region as silicone oil (secondary phase), while the remainder as culture medium (primary phase). The viscosity *μ* was inserted by setting the material property of silicone oil in the software. The viscoelastic stress term ***T*** was removed because it was considered as pure viscous liquid. Volume of fluid model and laminar flow was adopted. Then we run the simulation with 1 ms timestep. Please see the simulation procedure details in Appendix B.

## 3. Results

### 3.1. Spheroidization Result of Porcine Fetal Fibroblast and Its Simulation

The porcine fetal fibroblast was used in the experiments. 

**Figure 8:**
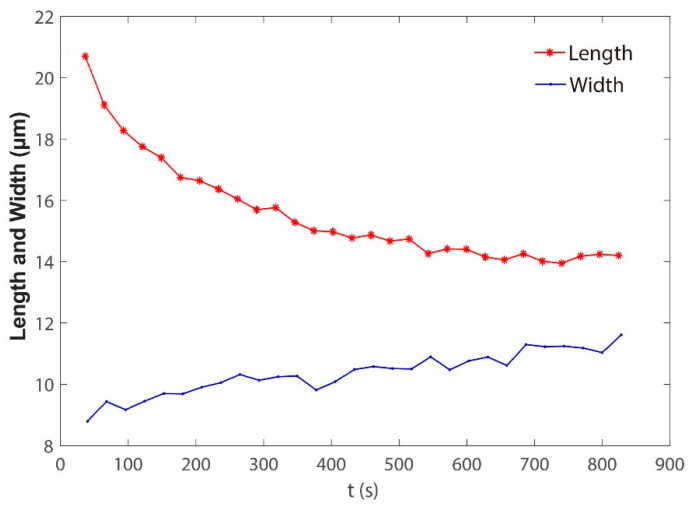
Variation of porcine fetal fibroblast length and width with time.

The typical images in the spheroidization process of porcine fetal fibroblast have been shown in Figure 1 and Figure 2 (Video S1). The length and the width changing process was shown in Figure 8. The whole spheroidization process took 15 min to reach a 90% width–length ratio.

**Figure 9:**
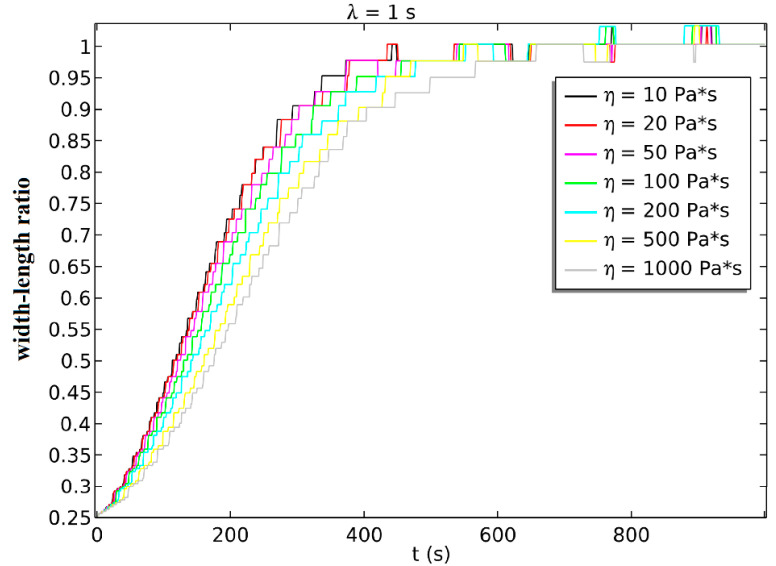
The simulated spheroidization process when *λ* = 1 and viscosity *η* changed.

We got the simulated width–length ratio at the condition that *λ* = 1 and changed viscosity *η*, as shown in Figure 9. Video S2 shows the simulated spheroidization process of porcine fetal fibroblast. The results show that a larger *η* will prevent the porcine fetal fibroblast from turning into a sphere, and the spheroidization time becomes longer.

**Figure 10:**
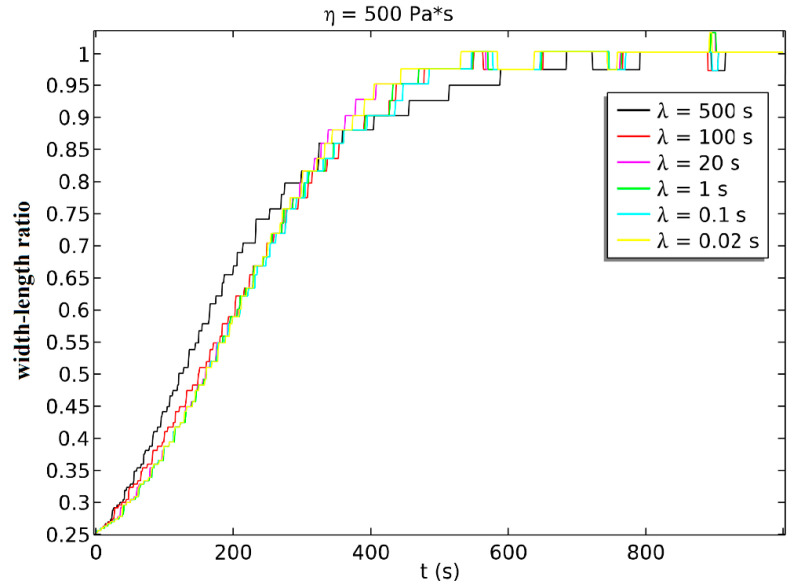
The simulated spheroidization process when *η* = 500 Pa·s and *λ* changed.

We got the simulated width–length ratio at the condition that *η* = 500 Pa·s and changed *λ*, as shown in Figure 10. The experimental results showed that the spheroidization process was more intense in the initial stage, but because the elasticity was smaller when *λ* was larger, the small elasticity will bring a lag effect in the later stage of spheroidization, which would make the later stage of spheroidization slow down significantly.

**Figure 11:**
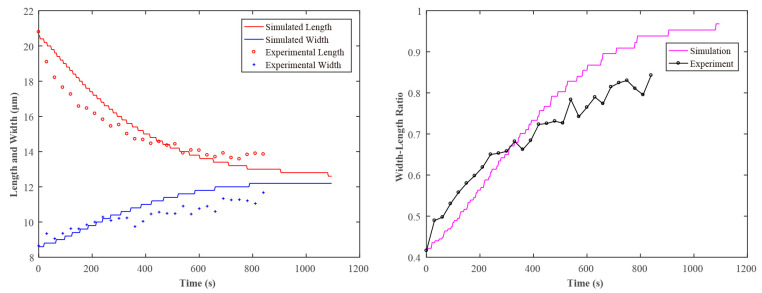
Variation of porcine fetal fibroblast length and width with time in the experiment and simulation.

Finally, by changing the values of *η* and *λ* in the simulation experiment, different curves of the spheroidization process of the simulated porcine fetal fibroblast were obtained. By comparing with the curves of the spheroidization process obtained in the real experiment, the elasticity and viscosity could be obtained. Figure 11 shows the length and width variation of porcine fetal fibroblast with time in the experiment and simulation. The viscosity *η* obtained in this experiment is 10 Pa·s and the elasticity *E* is 500 Pa. The magnitude of the results was in agreement with the measured results in [29].

### 3.2. Spheroidization Result of Silicone Oil and Its Simulation

**Figure 12:**
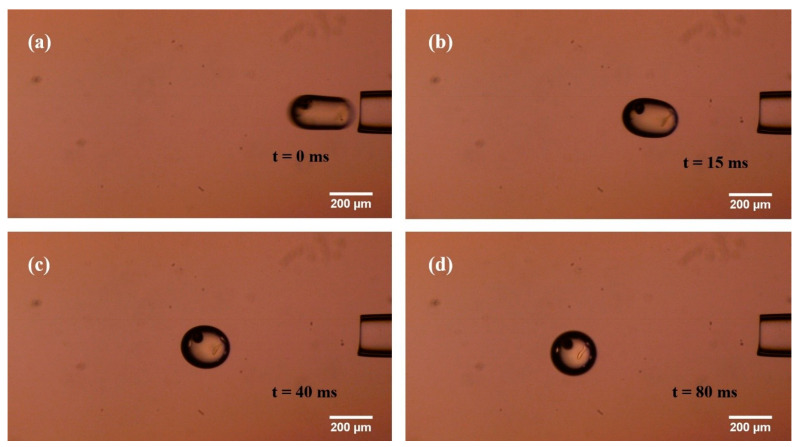
Typical images in the spheroidization process of silicone oil. (**a**–**d**) From silicone oil just coming out of the micropipette to silicone oil becoming a sphere.

Figure 12 shows the typical images in the spheroidization process of silicone oil. Video S3 shows this process of slowing down 100 times. Video S4 shows the simulated spheroidization process of silicone oil.

We defined the time span from the release to 95% width–length ratio as spheroidization time, which is denoted as *t_s_*. The *t_s_* was 80 ms in the experiment.

The viscosity and density of the silicone oil (Sigma-Aldrich) at 25 °C was 9.71 Pa·s and 0.971 g/mL. To simulate the spheroidization process of silicone oil, we also need to know the surface tension coefficient with culture medium of silicone oil. We measured the surface tension coefficient between silicone oil and culture medium by Du Noüy ring method [26], the coefficient was 0.024 N/m. The detail is shown in Appendix C. The method of detecting the contour of the silicone oil is shown in Appendix A.

**Figure 13:**
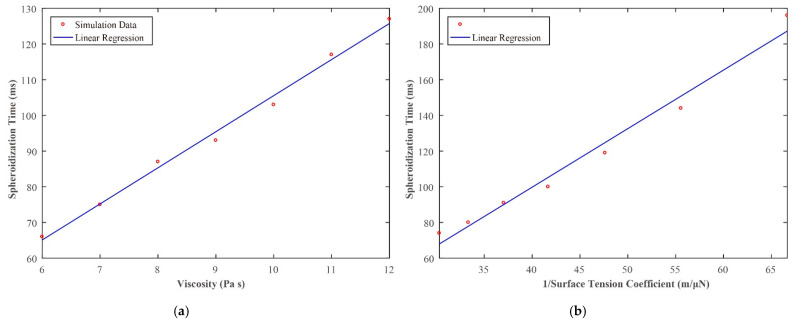
Simulation results: (**a**) spheroidization time increases linearly as viscosity increases. (**b**) Spheroidization time increases linearly as the surface tension coefficient increases.

Figure 13 shows the simulation results of silicone oil with different viscosities and surface tension coefficient. The R-square values of the fitted curves are 0.99 and 0.98. The results revealed that spheroidization time increases linearly as viscosity and the reciprocal of surface tension coefficient increases. The results showed that the spheroidization time *t_s_* was 100 ms in the simulation, which was similar to the real experiment (80 ms).

## 4. Discussion

We should know that the results measured in this paper were based on the bulk measurements, by which the cells were assumed as isotropic, homogeneous. However, in reality cells are very heterogeneous and contain organelles. We also need to measure the local force and dissipative gradients, as well as map them across the cell surface [34,35,36]. Considering the measuring efficiency, only two parameters are necessary to describe the cellular mechanics, so the bulk measurement is more appropriate.

We used the cells in the suspension state instead of adherent state in this paper. Because our method needs to aspirate to the whole cell into the micropipette, and it is difficult to aspirate the adherent cells into the micropipette because of the adhesion. Since the whole suspension cell was sucked into the micropipette, the cell spheroidization process was only related to the shape of the micropipette. The seal between the cell and the micropipette will not affect the spheroidization recording results, which can avoid the influence of seal in the micropipette aspiration method.

We performed the cell experiments three times and the simulations 12 times per cell. As the cells were collected from one batch, the experimental curves were very similar. The parameters in the simulation were set not very accurate (just integers), so the results of these three cells were the same. The measurement results may be significantly different among different cell types or different cell batches. Our future work will focus on measuring the viscoelasticity of different cell types and improving the simulation accuracy by adjusting the parameters more accurately.

We could see that the simulation results shown in Figure 10 do not overlap with the experiment results shown in Figure 7 exactly. We supposed that there were three reasons:

(1) The whole spheroidization process would take a tremendously long time in a in vitro environment, which would influence the viscoelasticity of the cell a lot. We only recorded the spheroidization process when the cells reached a 90% width–length ratio, which took about 15 min. Meanwhile, the simulation process recorded the whole spheroidization process. So, the experiment results and the simulation results could not overlap exactly.

(2) As mentioned above, the parameters in the simulation were not set very accurately (just integers), so the simulation results could not exactly fit the experiment results.

(3) The initial velocity was set to zero. This may have caused an initial acceleration stage from zero, while the non-zero initial shrink in velocity was found from the experiment. The problem was handled by running several steps in advance.

We know that if the viscous term of a viscoelastic body is increased, then it takes longer to get back to its original shape. Our results showed that a larger *η* will prevent the porcine fetal fibroblast from turning into a sphere, which could further verify the validity of our simulation results.

## 5. Conclusions

This paper presents a cell’s viscoelasticity measurement method based on the spheroidization process of a non-spherical shaped cell. We firstly introduced the process of recording the spheroidization process of porcine fetal fibroblast. We secondly built the viscoelastic model for simulating a cell’s spheroidization process. We simulated the spheroidization process of porcine fetal fibroblast and got the simulated spheroidization process. Then we got the elasticity (500 Pa) and viscosity (10 Pa·s) of porcine fetal fibroblast by identifying the parameters in the viscoelastic model. The results showed that the magnitude of the elasticity and viscosity were in agreement with those measured by a traditional method. To verify the accuracy of the proposed method, we imitated the spheroidization process with silicone oil, a kind of viscous and uniform liquid with determined viscosity. We did the silicone oil’s spheroidization experiment and simulated this process. The simulation results also fitted the experimental results well. 

## Figures and Tables

**Figure A1:**
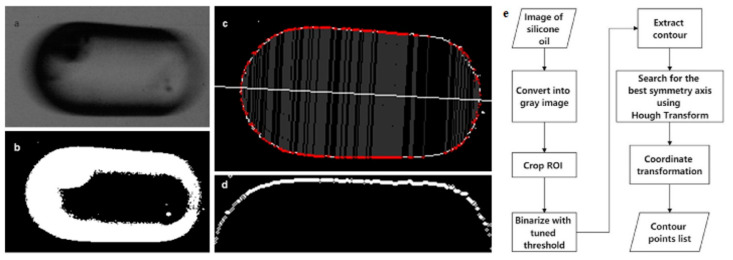
Image processing: (**a**) ROI of grayscale image; (**b**) binarized image; (**c**) axisymmetric contour; (**d**) upper side of contour; (**e**) flow chart of image processing.

**Figure A2:**
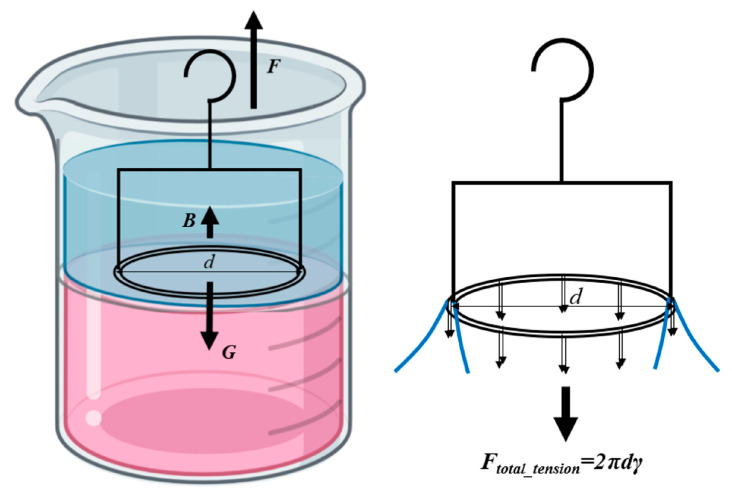
Du Noüy ring method to measure surface tension coefficient.

## Data Availability

The raw data supporting the conclusions of this article will be made available by the authors, without undue reservation.

## Supplementary Materials

The following are available online at https://www.mdpi.com/article/10.3390/s21165561/s1, Video S1: The spheroidization process of porcine fetal fibroblast (0 to 15 s: slowing down 5 times; 16 to 23 s speeding up 100 times). Video S2: The simulated spheroidization process of porcine fetal fibroblast. Video S3: The spheroidization process of silicone oil. Video S4: The simulated spheroidization process of silicone oil.

## Appendix A. Size Measurements of Porcine Fetal Fibroblast and Silicone Oil

In the initialization step of the simulation, we chose the image where the porcine fetal fibroblast and silicone oil were just out of the micropipette, then extracted its contour. The flow chart of image processing is shown in Figure A1. After the processes, we got a list of contour points where x axis was the axisymmetric axis. As the contour was symmetric, we used the upper half of it to conduct an axisymmetric simulation, which saved time and computational resources, and was more convenient to show the results. For the consecutive images, length and width were measured automatically for silicone oil and manually for fibroblast because of the low contrast ratio.

## Appendix B. Simulation Procedure

We used Ansys Student Fluent to simulate the spheroidization process. The Fluent software provides the volume of fluid (VOF) module for multiphase simulation such that we can simulate the surface tension between cells and culture medium. Because the velocities in such processes are small (of the order of 1 nm/s), the Reynolds number is small, so the laminar model was adopted. For the cell simulation, we used user-defined scalars (UDS) to introduce the viscoelastic stress term. Details of the simulation are described below.

**Geometry:** rectangle region of 16 × 10 μm^2^ for fibroblast and 500 × 200 μm^2^ for silicone oil;

**Mesh:** structural quadrilateral grids of 0.1 × 0.1 μm^2^ for fibroblast and 2 × 2 μm^2^ for silicone oil;

**Boundary conditions:**

left and top—pressure outlet with 0 Pascal gauge pressure;

right—symmetric for fibroblast and pressure outlet for silicone oil;

down—axisymmetric;

**Fluid filed settings:**

General: 2D axisymmetric;

Models panel: multiphase—VOF (Phase Interactions—Surface Tension), Viscous (Laminar);

**Materials**: The density and viscosity are set in this stage. In this paper, the values are: 1080 kg/m^3^ of density and 500 Pa s of viscosity for fibroblast, 9.71 kg/m^3^ of density and 9.71 Pa s of viscosity for silicone oil, 998.2 kg/m^3^ (the density of water at 20 °C) of density and 1.003 × 10^−3^ Pa s for surrounding liquid.

**Initialization and UDS equations:**

Ansys Student Fluent provides user-defined functions (UDF) for customizing fluid simulation. In this paper, a C code file utilizing predefined macros sets the initial fibroblast and silicone oil area, as well as introduces equations about viscoelastic stress ***T***. To add ***T*** into the momentum equation, four scalars were defined and added to the momentum equation by DEFINE_SOURCE macro. The usage of UDF can be found in official ANSYS Fluent UDF Manual. The initial velocities were set as zero.

For an arbitrary user-defined scalar ϕ, Fluent solves the Equation (A1).

(A1) $$\frac{\partial \rho \phi }{\partial t}+\frac{\partial }{\partial x_{i}}(\rho u_{i}\phi -\Gamma_{k}\frac{\partial \phi }{\partial x_{i}})=S$$

where *ρ* is the density, *u* is the velocity, *Γ* is the diffusion coefficient, *S* is the source term. The terms in the equation represent unsteady term, convective flux, diffusion and source from left to right. Expanding Equation (2) and using Einstein summation convention, we get Equation (A2):

(A2) $$\lambda \frac{\partial \tau_{ij}}{\partial t}+\lambda u_{k}\frac{\partial \tau_{ij}}{\partial x_{k}}-\lambda \tau_{ik}\frac{\partial u_{j}}{\partial x_{k}}-\lambda \tau_{kj}\frac{\partial u_{i}}{\partial x_{k}}+\tau_{ij}=\mu (\frac{\partial u_{i}}{\partial x_{j}}+\frac{\partial u_{j}}{\partial x_{i}})$$

The first term was added with DEFINE_UDS_UNSTEADY macro. The second term was added with DEFINE_UDS_FLUX macro. Other terms were added with DEFINE_SOURCE macro. It should be noticed that for 2D axisymmetric setup in this paper, the gradients along normal direction of the geometry plane were all zero. Therefore, only four scalars $\tau_{11},\tau_{12},\tau_{21},\tau_{22}$ were defined. Besides, a factor 2π should be multiplied for volume and area because of axisymmetric condition according to the manual.

**Solver**: 1 ms of timestep for silicone oil and 3 s for fibroblast. For each time step, iterate at most 100 steps with 0.001 as convergence absolute criteria.

**Notes:**

1. The quadrilateral grids are suggested for the meshing as it is more stable than triangular grids in the simulation. We suppose that it was because triangular grids cause larger curvature, which makes surface tension change dramatically within a local area.

2. The mesh size should not be too small, not only for the computation efficiency, but also for the convergence. When reducing the mesh size down to a certain scale, the simulation gets hard to converge. It is also supposed to be the result of large local surface tension.

## Appendix C. Surface Tension Coefficient Measurement of Silicone Oil

As is illustrated in Figure A2, when the interface between silicone oil and culture medium was flat and the platinum ring was on the interface, we have

(A3) $$F_{0}+B=G$$

where *F*_0_ is the external force in this case, *B* is the buoyancy, *G* is the total weight of platinum ring and its frame.

When we pull the platinum ring up, it stuck the interface, causing a circle of interface protruding (Figure A2), and an extreme thin film formed near the ring. Inner and outer interface both contributed to the surface tension, i.e.,:

(A4) $$F_{total\_tension}=2\pi d\gamma$$

where *γ* was the surface tension coefficient.

The pull is a quasi-static process, we can get

(A5) $$F_{m}+B=G+F_{total\_tension}$$

where *F_m_* was the maximum external force in the process. The change of *B* was negligible compared with change of external force, so we used the same notation *B*. Combining (A3), (A4) and (A5), we get:

(A6) $$\gamma =\frac{F_{m}-F_{0}}{2\pi d}=\frac{\Delta F}{2\pi d}$$

In this experiment, the diameter *d* was 14 mm, and we got that Δ*F* was 2.11 mN in average, so *γ* was 0.024 N/m.
